# Oxidative Stress-Mediated Neuroinflammation in the Pathophysiology of Schizophrenia

**DOI:** 10.3390/ijms262211139

**Published:** 2025-11-18

**Authors:** Mateusz Trubalski, Agnieszka Markiewicz-Gospodarek, Marta Żerebiec, Julia Poleszak, Miłosz Szczotka, Renata Markiewicz, Bartosz Łoza, Sylwia Szymańczyk

**Affiliations:** 1 Student Scientific Association at the Department of Normal, Clinical and Imaging Anatomy, Medical University of Lublin, 20-090 Lublin, Poland; mateusztrub@gmail.com (M.T.); zerebiecm22@gmail.com (M.Ż.); juliapoleszak3@gmail.com (J.P.); szczotkamilosz685@gmail.com (M.S.); 2 Department of Normal, Clinical and Imaging Anatomy, Medical University of Lublin, 20-090 Lublin, Poland; agnieszka.markiewicz-gospodarek@umlub.edu.pl; 3 Occupational Therapy Laboratory, Chair of Nursing Development, Medical University of Lublin, 20-081 Lublin, Poland; renata.markiewicz@umlub.edu.pl; 4 Department of Psychiatry, Medical University of Warsaw, 02-091 Warsaw, Poland; bartosz.loza.med@gmail.com; 5 Department of Animal Physiology, Faculty of Veterinary Medicine, University of Life Sciences in Lublin, 20-950 Lublin, Poland

**Keywords:** oxidative stress, neuroinflammation, schizophrenia, mental disorders, neurotransmitters

## Abstract

Schizophrenia is a complex neuropsychiatric disorder characterized by a diverse range of symptoms, including positive, negative symptoms such as alogia, anhedonia, avolition, and affective flattening, cognitive symptoms, and emotional symptoms as a separate domain. Emerging evidence suggests that oxidative stress and neuroinflammation play crucial roles in the etiopathogenesis of schizophrenia. The aim of this review is the interplay between oxidative stress—defined as an imbalance between reactive oxygen species (ROS) production and antioxidant defenses—and neuroinflammatory processes within the central nervous system. Studies indicate that elevated levels of oxidative markers and pro-inflammatory cytokines are commonly observed in individuals with schizophrenia, pointing to a potential pathophysiological link. The dysregulation of redox homeostasis may exacerbate neuroinflammatory responses, contributing to neuronal damage and the subsequent manifestation of psychiatric symptoms. Furthermore, genetic and environmental factors may interact with these biological processes, influencing individual susceptibility to schizophrenia. Understanding the mechanisms by which oxidative stress and neuroinflammation contribute to the development and progression of schizophrenia could pave the way for novel therapeutic strategies aimed at mitigating these pathological processes and improving patient outcomes.

## 1. Introduction

Schizophrenia is a psychiatric condition marked by negative symptoms and a core decline in various cognitive abilities, superimposed by positive symptoms, such as delusions and hallucinations and some other distorted or disorganized behaviors [1]. Currently, schizophrenia affects about 1% of the global population and correlates with a decrease in average lifespan up to 20 years [2]. The disease typically manifests with its exact onset of the first episode during late adolescence or early adulthood. It often follows a prodromal phase, and cognitive deficits may have been evident for many years before the onset [3]. Although schizophrenia tends to run in families, no single gene is responsible for the disease. On the contrary, it appears to be highly polygenic with a complex array of risk loci [4]. The combination of genetic and biopsychosocial factors such as maternal infection, obstetric complications, childhood trauma, or psychoactive substances contributes etiopathogenetically [4]. Currently, there are no causal (disease-modifying) drugs that directly target the underlying pathophysiological mechanisms of schizophrenia, as antipsychotics can reduce to some degree positive symptoms, but at most stabilize negative deficits, in which they are no more effective than other non-pharmacological techniques such as long-term rehabilitation therapy [1]. So, the etiopathogenesis of schizophrenia looks to arise from a complex interplay of genetic predispositions and environmental influences, with the area of correlation of these factors not clearly established. In this regard, intracellular and extracellular inflammatory processes are analyzed as one of several important contributing factors rather than the sole keystone [5,6]. These premises are based on modern in vivo, in vitro, and postmortem research, as well as on the reinterpretation of previous biochemical, neurotransmitter and neuroimaging studies [7,8,9].

Oxidation is the loss of electrons or the oxidation state increase, and simultaneously reduction is the gain of electrons or decrease of the oxidation state. This reaction is the core of all energetic intracellular processes. Specific and non-specific redox imbalance disturbances are increasingly identified in patients with schizophrenia [10,11,12].

These disruptions may constitute a central hub for the etiopathogenesis of schizophrenia with respect to both genetic and environmental risk factors. The aim of this work is to analyze the importance of the impact of oxidative stress and neuroinflammation on the onset and course of schizophrenia.

## 2. Methods

**Figure 1:**
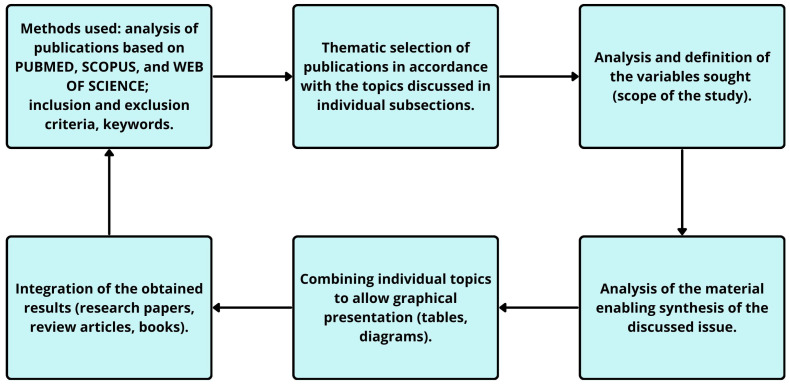
PRISMA 2020 Checklist [13].

**Figure 2:**
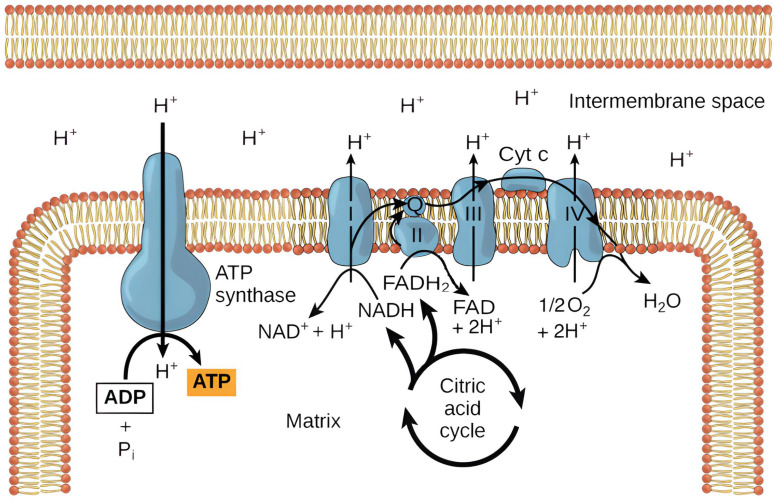
Summary of oxidative phosphorylation [14].

To explore the relationship between oxidative stress, neuroinflammatory mechanisms, and the etiopathogenesis of schizophrenia, we conducted a comprehensive literature review including clinical, experimental and systematic studies. The literature search was performed in three major databases: PubMed, Scopus, and Web of Science, focusing on the following key terms: schizophrenia etiopathogenesis OR (mental disorders AND etiology) OR (neuroinflammation AND oxidative stress AND reactive oxygen species); schizophrenia OR (ROS AND neurotransmitters); neurotransmitters OR (schizophrenia AND central nervous system); oxidative stress OR (schizophrenia AND HPA axis AND neurotransmitters). The literature search covered the years 2005–2025, ensuring that the review primarily reflects current knowledge in the field. In total, 158 references were included in the final bibliography. The inclusion criteria were (1) original and review publications published in peer-reviewed journals within this time frame, although a few seminal papers published before 2005 were also included because of their historical and conceptual importance for the development of this research area; however, they do not constitute leading items in the literature, (2) publications mainly in English (3) the use of structured database searches, which covered the main topic of the work in chronological order, in line with the topics of the individual subsections. After searching for a specific subject area consistent with the presented subchapter, those items that were relevant and important from the authors’ point of view and within the specific subject area were included in the review, while the remaining works were excluded. Due to the extensive subject area of the work, the authors limited the number of tables and figures to those necessary. A summary of the topic discussed in this paper is provided at the end of the paper and in the conclusions, as well as in Table 1, Figure 1 and Figure 2 The methodological process of literature selection and analysis, consistent with PRISMA 2020 checklist, is illustrated in Figure 1.

**Table 1:** ROS-induced damage to cellular components.

Cellular Component	Process Characterization
Lipids	The oxidative damage to lipids, especially in cell membranes, compromises membrane integrity and function. Lipid peroxidation not only alters the physical properties of membranes but also generates secondary reactive species that propagate damage throughout the cell. This disruption can impair membrane-bound signalling pathways and transport processes, leading to cellular dysfunction [15]. Recent studies in schizophrenia have shown elevated serum levels of oxidized LDL (ox-LDL) and its receptor LOX-1, both of which are products and mediators of lipid oxidation, implicating them as potential biomarkers of oxidative stress–driven neuroinflammation [16].
Proteins	Oxidative modifications of proteins can result in misfolding, aggregation, and loss of function. Enzymes with critical roles in metabolic pathways are particularly vulnerable, as oxidative damage can alter their catalytic efficiency. Moreover, oxidized proteins are often targeted for degradation by the proteasome system, leading to increased protein turnover and stress on the protein synthesis machinery [17].
Nucleic acids	DNA damage caused by ROS includes base modifications, single and double-strand breaks, and cross-linking. These lesions can interfere with transcription and replication processes, leading to genomic instability. The activation of DNA repair mechanisms, while essential for maintaining genomic integrity, consumes cellular resources and can induce further stress if repair processes are overwhelmed or defective [18,19].

## 3. Oxidative Stress

Oxidative stress is a disturbance of the internal pro- and anti-oxidative homeostasis. Excessive induction of pro-oxidant reactions and limited reactions neutralizing antioxidant processes leads to many biochemical and neuroanatomical disorders in various structures, at various levels. The basic level includes damage to cellular lipids, enzymes, proteins, carbohydrates and DNA [20]. It should be emphasized that the cells of the central nervous system (CNS) are extremely susceptible to the toxic effects of free radicals (ROS) since the brain has a high rate of oxidative metabolic activity, low level of antioxidant enzymes, high demand for oxygen and its high consumption, and an anatomical neuronal network extremely sensitive to disruptions. The share of easily oxidizable polyunsaturated fatty acids (PUFA) makes the brain more susceptible to oxidative stress [21].

In addition, oxidative stress increases the excess of autoxidative neurotransmitters, such as dopamine (DA) or noradrenaline (NA), the metabolism of which generates the production of large amounts of hydrogen peroxide (H_2_O_2_) and superoxide radicals (O_2_) [10] and causes the production of toxic amino acids, which contribute to cell proteolysis (degradation and breakdown). Of all the brain areas, the basal ganglia are particularly susceptible to damage induced by ROS, because they contain a high iron content [10,20,21,22].

Although the human body has a complex defense system that blocks the initiation of chain reactions, both enzymatic, such as superoxide dismutase (SOD), glutathione peroxidase (PpX) or catalase (CAT) [23], or non-enzymatic, such as glutathione (GSH), vitamin E, vitamin C, beta-carotene [24], excessive induction of ROS reactions may lead to disorders in compensatory mechanisms (neurons have exceptionally poor antioxidant protection, they have 50 times lower catalase content compared to e.g., hepatocytes, the content of reduced glutathione (GSH) is ~50% lower in neurons compared to other cells, e.g., ~5 μM in neurons compared to 10–11 μM in hepatocytes) [25]. This compensatory dysregulation at the basic level probably causes disturbances in the homeostasis associated with apoptotic processes, because they are intensified by reactive oxygen species.

Apoptosis (A) itself is a natural process of cell death, which is necessary to maintain the homeostasis of the organism, maintaining the internal balance between the formation of new cells and the elimination of unnecessary (autoreactive, mutated) ones. Disturbances in maintaining this homeostasis can lead to the development of various autoimmune, neoplastic, and degenerative diseases [26]. The process (A) can be induced by both internal and external factors. The internal (mitochondrial) pathway is activated by factors such as radiation, cytotoxic compounds, and high concentrations of reactive oxygen species [14]. The external pathway is induced by inflammatory or infectious factors, metabolic disorders.

The energy source of each cell is mitochondria, which create high-energy chemical bonds of adenosine triphosphate (ATP) through oxidative phosphorylation. Mitochondria consist of two membranes and two compartments: the outer membrane, the inner membrane, the intermembrane space, and the matrix. The outer membrane is rich in porins and proteins that create voltage-dependent anion channels (VDAC) which enable the transport of molecules with a mass of 5 kDa. The permeability of the membrane is controlled by proteins from the Bcl-2 family, and its marker enzyme is monoamine oxidase. The inner membrane, which forms numerous invaginations known as cristae, lacks porins and is impermeable to most ions and molecules. It contains components of the electron transport chain and ATP synthase, essential for energy production. The transport of nucleotides such as ATP and ADP across the inner membrane occurs via the adenine nucleotide translocator (ANT) [20,21,27].

The marker of the inner membrane is the enzyme cytochrome C oxidase. The proteins of the inner membrane fulfill important functions—they form the respiratory chain, synthesize ATP and enable the transport of selected metabolites within the matrix. During oxidative phosphorylation, electrons from reduced carriers (NADH and FADH2) are transferred through complexes I-IV of the electron transport chain to oxygen, generating a proton gradient across the inner membrane. The resulting electrochemical potential drives ATP synthesis by complex V (ATP synthase), which converts ADP and inorganic phosphate into ATP as protons flow back into the matrix. This process couples oxidation and phosphorylation, ensuring efficient cellular energy production. Intermembrane proteins such as cytochrome C and procaspase also participate in this process (Figure 2) [14,20,21,28]. In the case of schizophrenia, the normal pattern of chemical reactions is limited. At the cellular level, there are dysfunctions in the functioning of mitochondria and metabolic dysfunctions, which result in abnormal neuronal formation, abnormal myelination, impaired production of neurotransmitters, and abnormal reorganization of synaptic connections. Disturbed homeostasis in connection with these reactions causes a deficit in maintaining membrane potentials, which in turn prevents the proper sensitivity of neurons to oxidative stress [20].

This process is exacerbated by Ca^2+^ imbalance, which additionally increases oxidative stress in neurons by blocking the Ca^2+^ pump of the endoplasmic reticulum. This leads to excessive concentration of Ca^2+^ ions in the cytoplasm of the neuron, which causes disorders in the release of neurotransmitters in synaptic terminals, abnormal activity of the N-methyl-D-aspartate receptor (NMDAR) and abnormal processes related to neurogenesis and synaptic plasticity [14,29]. Cascading processes at the basic level related to the effect of oxidative stress on cells are probably the cause of disorders related to the apoptosis process. Decreased energy efficiency of the cell probably leads to its aging and induction of degenerative processes.

## 4. Impact of Oxidative Stress on Cellular Metabolism

As mentioned before oxidative stress refers to the imbalance between the production of reactive oxygen species and the cell’s ability to detoxify these reactive intermediates or repair the resulting damage. This imbalance leads to the disruption of various metabolic pathways and normal cellular functions through the oxidative damage of lipids, proteins, and nucleic acids (Table 1).

### 4.1. Disruption of Metabolic Pathways

(a) Glycolysis and the tricarboxylic acid (TCA) Cycle: Oxidative stress significantly affects carbohydrate metabolism, including glycolysis and the TCA cycle. ROS can modify key enzymes such as glyceraldehyde-3-phosphate dehydrogenase (GAPDH) and aconitase, leading to altered enzyme activities and metabolic fluxes. For example, the oxidation of GAPDH results in decreased glycolytic flow, while aconitase inactivation disrupts the TCA cycle, leading to reduced ATP production and energy imbalance in cells [30,31];
(b) Lipid Metabolism: Lipid peroxidation is a primary consequence of oxidative stress, were ROS attack polyunsaturated fatty acids in cell membranes, generating lipid peroxides. This process alters membrane fluidity and permeability, affecting membrane-bound enzymes and receptors. Additionally, lipid peroxidation products, such as malondialdehyde (MDA), can form adducts with proteins and DNA, further impairing cellular functions [32,33]. Additionally, recent evidence indicates that oxidized low-density lipoprotein (ox-LDL) and its receptor LOX-1 play a significant role in oxidative stress–related neuroinflammatory pathways. In a case–sibling–control study, serum ox-LDL and LOX-1 levels were markedly elevated in schizophrenia patients compared to both healthy controls and unaffected first-degree relatives. Interestingly, ox-LDL levels were also higher in relatives than in controls, suggesting a possible endophenotypic marker of vulnerability. Activation of LOX-1 by ox-LDL is known to trigger pro-inflammatory signaling cascades, adhesion molecule expression, and ROS generation via the AMPK/PKC/NADPH oxidase pathway, which may exacerbate neuronal damage and blood–brain barrier dysfunction in schizophrenia [16];
(c) Protein Metabolism: Proteins are susceptible to oxidative modifications by ROS, which can lead to the formation of carbonyl groups, disulfide bonds, and cross-linked aggregates. These modifications often result in loss of enzymatic activity, altered protein structure, and impaired protein-protein interactions. For instance, oxidative damage to enzymes involved in amino acid metabolism can disrupt the synthesis and degradation of proteins, affecting overall cellular homeostasis [34];
(d) Nucleotide Metabolism: ROS can induce oxidative modifications in nucleotides, leading to the formation of 8-oxoguanine and other oxidized bases. These modifications can cause mutations during DNA replication, disrupt gene expression, and activate DNA repair pathways. In severe cases, the accumulation of DNA damage can trigger cell cycle arrest, pathological apoptosis, or carcinogenesis [35].

### 4.2. Mitochondrial Dysfunction

Mitochondria play a crucial role as both a source and target of reactive oxygen species. ROS are primarily produced at complexes I and III of the electron transport chain, where electrons can prematurely react with oxygen to form superoxide (O2•−) [36,37]. Oxidative damage to mitochondrial components, such as mtDNA, can lead to mutations that disrupt the encoding of proteins essential for the respiratory chain’s function. This, in turn, decreases the efficiency of ATP production, resulting in cellular energy deficiency [38]. Additionally, oxidative modifications of electron transport chain proteins can impair electron flow, increasing ROS production and creating a vicious cycle of oxidative stress and mitochondrial dysfunction [39]. Lipid peroxidation in the mitochondrial membrane disrupts its integrity, affecting the mitochondrial membrane potential and further hindering ATP production [40]. These changes contribute to metabolic dysregulation, as cells cannot efficiently produce energy, impacting their functions and leading to various pathologies.

### 4.3. Altered Redox Balance

Oxidative stress disrupts the cellular redox balance, significantly affecting the activity of redox-sensitive enzymes and signaling pathways. When the production of reactive oxygen species surpasses the cell’s antioxidant defenses, it results in the oxidation of crucial biomolecules, including lipids, proteins, and DNA. Redox-sensitive enzymes, which depend on specific oxidation states for their proper function, can become either inactivated or excessively activated under oxidative stress. This disruption in enzyme activity can lead to alterations in key signaling pathways, impacting cellular functions such as proliferation, apoptosis, and metabolism [41,42]. Antioxidants are essential in counteracting ROS-induced damage by neutralizing these reactive species and maintaining redox homeostasis. They achieve this by donating electrons to stabilize ROS, preventing the oxidative damage of cellular components, and ensuring the proper functioning of redox-sensitive systems. By maintaining the balance between oxidants and antioxidants, these compounds help preserve cellular integrity and function, thereby mitigating the adverse effects of oxidative stress on cellular processes [43,44].

An example of disrupted cellular homeostasis is the inappropriate distribution of hydrogen sulfide, produced during metabolic processes in which sulfur is transferred from homocysteine to cysteine. Homocysteine, a sulfur-containing amino acid, plays an important role in the metabolism of methionine, the synthesis of nucleic acids and phospholipids. Its metabolism is conditioned by normal metabolism associated with B vitamins and folic acid. Enzymatic deficiencies of the pathway of homocysteine metabolism are the cause of various diseases, the common feature of which is an increase in the concentration of this amino acid in body fluids. Homocysteine is synthesized from methionine, which, under the influence of methionine adenosyltransferase, is converted to S-adenosylmethionine and then to S-adenosylhomocysteine.

S-adenosylmethionine is an important donor of methyl groups; it provides the methyl radical for the synthesis of DNA, RNA, methylation of proteins, lipids, and creatinine synthesis. The metabolism of homocysteine involves its conversion to cystathion and then to cysteine, in which the enzymes β-cystathionine synthase (CBS) and γ-cystathionine synthase (CSE) participate. Homocysteine is metabolized mainly in the liver and kidneys. In these organs, there is a high activity of the primary enzymes that metabolize homocysteine, i.e., β-cystation synthase, betaine-homocysteine methyltransferase and methionine adenosyltransferase. The liver metabolizes much of the protein-bound homocysteine, while in the kidneys, small-molecule homocysteine disulfides are broken down and excreted in the urine. The leading way of removing homocysteine from the body is through transsulfuration reactions, occurring primarily in the liver and kidneys. Another way is the reverse conversion of homocysteine to methionine, which can occur by remethylation. There are two remethylation pathways: (1) a reaction involving vitamin B12 and folic acid; this reaction is considered the main pathway for the reverse conversion of homocysteine to methionine, (2) activation of betaine-dimethylglycine methyltransferase (conversion of homocysteine to methionine with the involvement of choline and betaine) [44,45].

Control of the transculturation pathway is important for maintaining optimal cellular function, as it modulates important physiological processes based on normal levels of hydrogen sulfide produced during biochemical metabolism. Both too much and too little H2S can have detrimental consequences for cells. The potential role of H2S in the pathogenesis of schizophrenia seems to be confirmed by studies conducted on an animal model of schizophrenia, which reflect deficits in sensorimotor gating, considered a biological feature of schizophrenia [46]. The consequence of dysregulation of the transculturation pathway (aberrant redox homeostasis) can also be the development of disorders, such as vascular dysfunction, Huntington’s disease, and aging, the cause of which depends on reduced expression of CSE, reduced production of cysteine (Cys) and H_2_S, and elevated levels of oxidative stress [47,48]. Physiological concentrations of H_2_S and its metabolites have been shown to enhance N-methyl-D-aspartate (NMDA) receptor function and induce long-term potentiation (LTP) in the hippocampus, while high concentrations of H_2_S are toxic and inhibit synaptic transmission in brain structures [49,50].

### 4.4. Dysregulated Metabolic Pathways

Oxidative stress significantly impacts metabolic pathways, such as glycolysis, the tricarboxylic acid (TCA) cycle, and fatty acid metabolism. This influence is exemplified by the oxidative modifications of key enzymes within these pathways, which disrupt their normal functions, leading to metabolic alterations within the cell. For instance, in glycolysis, enzymes like glyceraldehyde-3-phosphate dehydrogenase (GAPDH) are susceptible to oxidative inactivation, impairing glucose metabolism [42,51,52]. Similarly, oxidative stress can affect enzymes in the TCA cycle, such as aconitase, leading to diminished energy production and accumulation of reactive intermediates [36]. Additionally, oxidative damage to enzymes involved in fatty acid metabolism, such as carnitine palmitoyltransferase I (CPT1), can disrupt lipid metabolism and contribute to cellular dysfunction [40]. These ROS-mediated modifications initiate metabolic reprogramming, characterized by alterations in metabolic flux and substrate utilization. Cells under oxidative stress may shift their metabolism towards alternative pathways, such as the pentose phosphate pathway (PPP) or aerobic glycolysis, to meet their energy demands and maintain redox homeostasis [53,54]. However, this metabolic adaptation often comes at the expense of cellular function, as excessive ROS production and metabolic dysregulation can lead to cellular damage and dysfunction.

### 4.5. Consequences for Cellular Health

Oxidative stress-induced metabolic alterations lead to several critical consequences, including impaired energy production, increased susceptibility to apoptosis, and promotion of inflammatory responses. Impaired energy production results from oxidative damage to mitochondrial enzymes, which disrupts ATP synthesis and leads to cellular energy deficits. Elevated ROS levels trigger pathological apoptosis by activating pro-apoptotic signaling pathways, thereby increasing cell death rates [55]. Furthermore, oxidative stress enhances inflammatory responses by activating nuclear factor-kappa B (NF-κB) and other inflammatory mediators [56]. These alterations have significant implications for various medical conditions [57,58].

**Figure 3:**
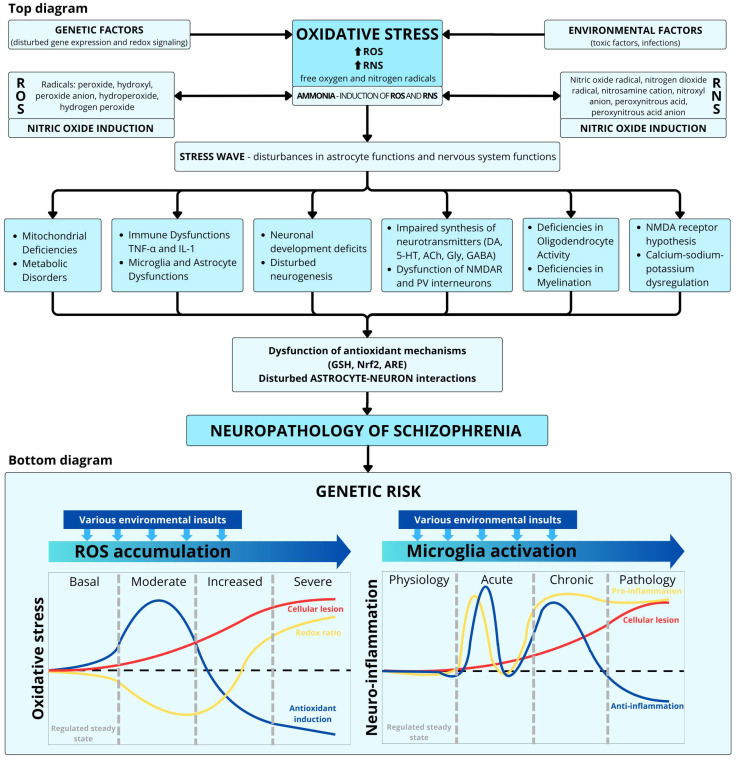
Top diagram: Evolution of redox regulation and interaction with neuroinflammation.

There is now, however, a clear consensus that schizophrenia is largely a neurodevelopmental disease, not a neurodegenerative one [59]. On the other hand, the neurodevelopmental theory does not exclude the presence of neurodegenerative phenomena, including common signs of neurotoxicity, white and gray matter structural disintegration leading to dysconnectivity and neurocognitive deficits, and finally the fact of patients’ accelerated aging [60]. In schizophrenia, a critical imbalance of excitatory glutamatergic neurons and inhibitory GABA interneurons may lead to glutamate-dependent over-pruning via apoptotic loss of dendritic spines [61]. An increased susceptibility to apoptosis was detected in cultured somatic cells from patients with schizophrenia [62]. Moreover, those apoptotic mechanisms did not affect non-schizophrenia psychotic patients. An increased serum level of apoptotic circulating nucleosomes was found in patients with negative schizophrenia [63]. Similarly, the caspase pathway, fundamental for apoptosis, is overactivated in schizophrenia, as the caspase-3 and caspase-9 gene expression serum levels were significantly increased in patients with schizophrenia [64]. The schizophrenia hypothesis, targeting apoptosis, can conceptualize pharmacological interventions during the prodromal phase and the transition to psychosis [61]. Figure 3 showed the evolution of redox regulation and interaction with neuroinflammation.

In psychiatry, impairment of critical processes results from complex interactions between genetic and environmental risk factors during brain development. The transition from a physiological state to a severe pathological state depends on the intensity of oxidative stress and microglia activation and the imbalance between compensatory antioxidant and anti-inflammatory systems. Legend: ROS—reactive oxygen species; RNS—reactive nitrogen species; IL-1—interleukin 1; TNF-α—tumor necrosis factor; DA—dopamine; 5-HT—serotonin; Ach—acetylcholine; Gly—glycine; Glu—glutamic acid; GABA—gamma-glutamic acid; GSH—glutathione; Nrf 2—antioxidant factor; ARE—antioxidant protein; N-methyl-D-aspartate receptor—receptor for glutamate; parvalbumin (PV) interneurons. Bottom diagram: The evolution from basal physiologically regulated steady state to severe pathological state depends on the intensity of the induced oxidative stress (OxS) or microglia activation but also on an imbalance of the compensatory anti-antioxidant and anti-inflammatory systems. Legend: blue curve shows the path of a reactive oxygen species (ROS)-induced redox-sensitive parameter (e.g., the activity of an antioxidant enzyme). Red curve indicates the evolution of ROS-modified molecules (e.g., oxidized lipids, proteins, or nucleic acids). Yellow curve shows the path of the redox ratio; the blue and yellow curves show the dynamic evolution of anti- and proinflammatory factors, respectively. Both are tightly regulated under physiological conditions but tip toward proinflammation in chronic and pathological states: the red curve shows the evolution of brain cellular and molecular consequences of neuroinflammation [65].

## 5. Disturbance in Neurotransmission

Neurotransmitters are fundamental building blocks of neuronal signaling pathways that regulate the complex homeostasis of the organism by transmitting signals between neurons, neurons and muscles or acting on the electrochemical state of other cells [29,66]. Neurotransmitters can be classified into two categories: small-molecule transmitters and neuropeptides. Small-molecule transmitters, such as dopamine and serotonin, exert rapid, direct effects on neighboring cells. In contrast, neuropeptides, such as oxytocin, show a complex action. Neurotransmitters are important for the integral balance of an individual’s mental and physical health, and abnormalities in their regulation may be a predisposing factor for mental disorders [66].

### 5.1. Dopamine

It is the most classical monoamine (dopamine) instability concept of psychosis [67,68]. The dopaminergic concept of schizophrenia is based on two main observations: the psychedelic/hallucinogenic effect of hyperdopaminergic substances and ex iuvantibus the beneficial effect of antipsychotics, predominantly antidopaminergic [69,70].

Various genetic studies, from classical single nucleotide polymorphism to genome-wide association studies, consistently demonstrate associations of the dopamine receptor D2 (DRD2) gene with leading pathophysiological hypotheses of schizophrenia [Schizophrenia Working Group]. On the other hand, even the largest genome-wide association studies, considering all risk variants, can predict case-control status only with low sensitivity and specificity, as currently only 3.4% of cases are explained by genome-wide significant loci [71].

The dopamine concept also comes into crisis as the antipsychotic efficacy is not proportional to dopamine antagonism. On the other hand, the concept was partially revitalized with the introduction of atypical antipsychotics, with only limited antagonism or even with partial agonistic dopaminergic action [72,73].

The dopaminergic hyperexcitability model, based on repeated amphetamine administration, reflects well the symptoms of schizophrenia, such as reduced pre-impulse inhibition, stereotypic behavior and impaired cognitive flexibility and attention. Amphetamine induces dopamine release, and its effects can be attenuated by dopamine antagonists, suggesting a role for dopamine in psychotic symptoms [74].

Dopamine transmission disorders, including schizophrenia, are associated with the umbrella concept of hypofrontality, which means a state of decreased cerebral blood flow (CBF) in the prefrontal cortex, associated with reduced oxygen and glucose consumption [68]. This condition determines negative, depressive and some cognitive symptoms in schizophrenia. On the other hand, hypofrontality is usually combined with symptoms of hyperfrontality, i.e., positive symptoms, distorted behavior and some other cognitive symptoms in schizophrenia [75,76]. Although it is the symptoms of dopaminergic hyperfrontality that lead to the diagnosis of schizophrenia, they only mask hypofrontality, which precedes the clinically overt onset of the disease for years and ultimately dominates the long-term course of schizophrenia.

Evidence of prefrontal hypodopaminergic comes especially from studies using positron emission tomography, which showed reduced blood flow in the frontal cortex directly correlated with low levels of dopaminergic system (DA) metabolites in the cerebrospinal fluid in patients with schizophrenia. In addition, animal studies have provided direct evidence linking prefrontal hypodopaminergic to subcortical hyperdopaminergic. Damage to dopamine neurons in the prefrontal cortex (PFC) led to increased levels of DA and its metabolites and D2 receptor density in the striatum, while administration of DA agonists to the PFC reduced levels of DA metabolites in the striatum [76,77].

The elevated dopamine synthesis capacity in the striatum, as measured by fluorodopa uptake into dopaminergic terminals, observed in studies, is consistently observed in patients with schizophrenia and shows a correlation with the severity of psychotic symptoms [70]. This is also supported by a study by Brugger et al., in which a meta-analysis of variance was conducted to examine interindividual variability in striatal dopaminergic function in patients with schizophrenia and healthy controls [78].

Dopamine metabolism leads to the formation of ROS. Chowdari’s [79] genetic study concluded that schizophrenia patients may have a reduced ability to produce adequate antioxidant defenses, including glutathione synthesis [64]. Reduced levels of glutathione (GSH) in the cerebrospinal fluid and prefrontal cortex were observed in patients. GSH deficiency can cause peroxidation of membrane lipids and microdamage around dopaminergic endings, leading to loss of neural connectivity and dysconnectivity syndrome.

Dopamine can be oxidized non-enzymatically to o-quinone form or enzymatically by monoamine oxidase to 3,4-dihydroxyphenylacetaldehyde, which leads to the production of H_2_O_2_ [10]. Dopamine non-enzymatic oxidation is probably the predominant source of elevated hydrogen peroxide concentration in the brain [80]. As a result of dopaminergic dysfunction, hyperglutamatergic neurotransmission develops, leading to late-onset brain damage. Another adverse interaction of dopaminergic activity occurs with nitric oxide, whose serum level is increased in schizophrenic patients, resulting in peroxynitrite production, a neurotoxic agent [10,72].

Hypo- or hyperfrontality is not related exclusively to dopamine transmission disorders. The underlying mechanism remains unclear, but it has been speculated that it may result from N-methyl-D-aspartate receptor (NMDAR) underactivation and parvalbumin (PV) neuronal dysfunction, leading to disinhibition of dopamine neurons in the mesostriatal pathway [77]. Post-mortem analysis of brain tissue, cerebrospinal fluid studies and pharmacological tests indicate a deficit of 5-HT function in the cerebral cortex in patients with schizophrenia. Serotonin receptors have been shown to modulate dopaminergic function, and serotonin can both inhibit and stimulate dopamine release in the striatum [81,82]. Yamamoto analyzed how different drugs that modulate schizophrenia symptoms affect the dopaminergic (DA) and noradrenergic (NE) systems. Evidence suggests that positive symptoms of schizophrenia are associated with hyperactivity of the NE system, whereas negative symptoms are associated with hypoactivity of this system [83]. Grima et al. [84] tested this hypothesis by examining the effect of DA on cultured cortical neurons with low GSH levels. They found that DA alone reduced the GSH pool level by 40%, suggesting that this effect is due to a direct coupling of DA semiquinone/quinone to GSH.

### 5.2. Serotonin

Serotonin, or 5-hydroxytryptamine (5-HT), is a vital signaling molecule involved in various central and peripheral functions in the human body. Within the central nervous system (CNS), serotonin acts as a neurotransmitter essential for numerous brain functions, influencing anxiety and behavior [85]. Additionally, it plays a role in the neuronal regulation of gut motility and the secretion of intestinal fluids. Beyond its role in neuronal communication in the CNS and enteric nervous system (ENS), serotonin also affects peripheral tissues. It is involved in several non-neuronal processes, including bladder function, respiratory drive, blood clotting, vascular tone, immune response, and intestinal inflammation [86]. The synthesis of serotonin starts with the conversion of tryptophan to 5-hydroxytryptophan (5-HTP) by the enzyme tryptophan hydroxylase (TPH). Subsequently, 5-HTP undergoes decarboxylation to form serotonin (5-HT) [87]. There are two isoforms of the enzyme TPH: TPH1 and TPH2. TPH2 is primarily found in the raphe nuclei of the brainstem, whereas TPH1 is found in both peripheral tissues and the central nervous system, including the pineal gland, thymus, spleen, and enterochromaffin cells in the gastrointestinal tract. Although serotonin plays crucial roles in the CNS, only 2% of the body’s serotonin is synthesized by serotonergic neurons in the brain. In contrast, approximately 90% of serotonin is produced by enterochromaffin cells in the gastrointestinal tract. Additionally, some studies have shown that other epithelial cells, such as hepatocytes and renal proximal tubule cells (RPTCs), are also capable of synthesizing serotonin [88].

The serotonin pathway includes its synthesis, transport, signal transmission in postsynaptic cells, and breakdown. 5-HT is stored in secretory granules by the solute carrier SLC18A1, also referred to as the vesicular monoamine transporter VMAT1, until released into the synaptic cleft. Upon presynaptic cell excitation, serotonin is released and binds to 5-HT receptors (HTR) [88]. Two distinct populations of serotonin binding sites, 5-HT1 and 5-HT2, have been identified in the rat brain. There are at least 20 cloned subpopulations of 5-HT receptors. The 5-HT1 receptor group includes five subtypes: 5-HT1A, 5-HT1B, 5-HT1D, 5-HT1E, and 5-HT1F, which are structurally similar in humans by 40–63%. These receptors are mainly, but not exclusively, associated with Gi/G0 proteins and inhibit the production of cAMP. The 5-HT2 receptor class consists of three subtypes: 5-HT2A, 5-HT2B, and 5-HT2C, which share 46–50% structural homology. They are predominantly linked to Gq11 proteins, which increase inositol trisphosphate hydrolysis and intracellular Ca^2+^ concentration [89]. This class is the primary excitatory receptor subtype among serotonin’s G-protein coupled receptors, although 5-HT2A may also have inhibitory effects in specific regions, such as the visual cortex and the orbitofrontal cortex [90]. The remaining 5-HT is reabsorbed into the presynaptic cell by SLC6A4, which is a solute carrier also known as 5-HT transporter (5-HTT) and stored in secretory granules or broken down by monoamine oxidases MAOA and MAOB [88]. Multiple postmortem studies have provided evidence supporting the involvement of altered serotonin (5-HT) transmission in the pathophysiology of schizophrenia. Consistently, findings indicate reduced density of cortical 5-HT transporters and increased binding of cortical 5-HT1A receptors in individuals with schizophrenia [81]. In a study conducted by Muguruza et al., the density of 5-HT2A receptors was examined in postmortem brain samples from individuals with schizophrenia, untreated and treated, as well as suicide victims with other psychiatric disorders, compared to matched controls in terms of gender and age. The research found no significant change in 5-HT2A receptor density among suicide victims with other psychiatric disorders when compared to controls. However, individuals with schizophrenia exhibited an increased active conformation of 5-HT2A receptors in the frontal cortex, suggesting a link between elevated 5-HT2A receptor density and schizophrenia rather than suicide. Furthermore, individuals treated with antidepressants showed a downregulation of 5-HT2A receptors [91]. Alongside direct evidence from postmortem studies, pharmacological interventions targeting 5-HT transmission have yielded data suggesting the role of serotonin in mediating symptoms of schizophrenia [81].

### 5.3. Acetylcholine

Acetylcholine (ACh) is a key neurotransmitter found in both vertebrates and invertebrates, serving as the principal transmitter within the cholinergic system (ChS) [92,93]. Its discovery is attributed to Henry Hallett Dale, while Otto Loewi later emphasized its role in chemical conductivity [92]. Acetylcholine is crucial for modulating neuronal activity in both the peripheral and central nervous systems [94]. ACh within the brain modulates neuronal excitability, impacts synaptic transmission, fosters synaptic plasticity, and orchestrates the coordinated firing of neuron groups. This leads to the alteration of neuronal network states across the brain, influencing their reactions to both internal and external stimuli, reflecting its classical function as a neuromodulator [95]. Two main cholinergic pathways in the brain can be distinguished: the magnocellular basal forebrain cholinergic system (ChS) and the brainstem cholinergic system. The ChS comprises the medial septal nucleus, the nucleus basalis of Meynert, and the vertical and horizontal limbs of the diagonal band of Broca, as well as the substantia innominata. This system projects widely to various brain regions, including the neocortex, entorhinal cortices, hippocampus, basolateral amygdala, and olfactory bulb. In contrast, the brainstem cholinergic system, which includes the pedunculopontine nucleus and the laterodorsal pontine tegmental nucleus, primarily projects to thalamic structures and basal forebrain regions.

ACh synthesis occurs within nerve terminals via the enzymatic activity of choline acetyltransferase (ChAT), which combines acetyl coenzyme A (CoA) with choline (Ch) to produce the neurotransmitter. Following release and synaptic function, ACh molecules undergo breakdown by acetylcholinesterase (AChE) into acetate and choline. Any ACh molecules not released into the synaptic cleft are stored in granules. The presence of ChAT within neurons signifies their reliance on ACh as a neurotransmitter [43]. Acetylcholine receptors (AChRs) are neurotransmitter receptors categorized into two primary subtypes: ionotropic nicotinic acetylcholine receptors (nAChRs) and metabotropic muscarinic acetylcholine receptors (mAChRs) [96]. The mAchRs are part of the G protein-coupled receptor (GPCR) superfamily, also known as metabotropic receptors. When a ligand binds to a GPCR, it activates and releases the associated G protein, which then triggers a cascade involving primary effector proteins and secondary messengers, depending on the G protein type. The mAChRs family comprises five subtypes, M1 through M5, which are divided into two groups. The ‘M1-like’ receptors, including M1, M3, and M5, are located post-synaptically and are coupled with Gq/G11 proteins, leading to excitatory effects. The ‘M2-like’ receptors, comprising M2 and M4, are found both pre- and post-synaptically and are coupled with Gi/Go proteins, resulting in predominantly inhibitory effects [97,98]. The nAChR family consists of ligand-gated ion channel receptors, also known as ionotropic receptors. These excitatory receptors are found both pre- and post-synaptically in the CNS and play a role in modulating the release of various neurotransmitters. Dysfunction in the nicotinic cholinergic system has been linked to the neuropathology of numerous diseases, including schizophrenia [97]. In neuropsychiatric drug discovery programs, the α4β2 heteromeric and α7 homomeric nAChRs are the most frequently targeted nicotinic acetylcholine receptor subtypes, as they are the most prevalent in the mammalian brain. Among these, the α7-nAChR is more commonly targeted for addressing negative deficits in schizophrenia. This focus is partly due to several factors, including postmortem evidence showing α7-nAChR deficits in the frontal cortex and hippocampus of individuals with schizophrenia, and linkage analysis pointing to chromosome 15q14, where the α7-nAChR gene is located. Polymorphisms in the core promoter of the α7-nAChR gene have been linked to reduced inhibition of the P50 sensory gating evoked response to repeated auditory stimuli in patients with schizophrenia, indicating these gating abnormalities. Deficits in α7-nAChR may also contribute to issues with smooth pursuit eye movements, sustained attention, and other cognitive and negative domains in schizophrenia [99].

### 5.4. Gamma-Aminobutyric Acid

One of the leading hypotheses for the development of schizophrenia is a loss of gamma-aminobutyric acid (GABA) signaling [100]. Gamma-aminobutyric acid is the primary neurotransmitter responsible for inhibition in the mammalian brain [101]. GABA is a non-protein amino acid generated through the α-decarboxylation of L-glutamic acid, a reaction catalyzed by the enzyme glutamate decarboxylase [102,103]. It is primarily located in the brain, but both GABA and its receptors are also present in the peripheral system, endocrine system, and various non-neural tissues, where it contributes to oxidative metabolism. GABA interacts with three types of receptors: alpha (A), beta (B), and gamma (C), all of which are found on the postsynaptic membrane. GABA-A and GABA-C receptors are ligand-gated ion channels, whereas GABA-B receptors are G protein-coupled receptors. GABA-A receptors facilitate fast synaptic transmission, while GABA-B receptors facilitate slow synaptic transmission. GABA-A receptors are linked to seizure threshold, anxiety, and panic, while GABA-B receptors are connected to memory, mood, and pain. Although GABA-C receptors have been identified, their physiological role remains unclear [103].

Microdialysis studies in humans and rodents have shown that increased glutamate and GABA release correlates with improved cognitive function [104]. In contrast, functional magnetic resonance spectroscopy shows that in schizophrenia, the increase in the ratio of glutamate to GABA that is induced by cognitive experiences is reduced [105]. Glutamate deficiency negatively affects glutathione and NO synthesis, which mediates schizophrenia symptoms [106].

There is a hypothesis that a deficit of GABA, whose transmission occurs via GABA receptors, is the primary cause of cortical circuit dysfunction in schizophrenia, while cognitive deficits result from altered gamma oscillation circuitry [107]. This hypothesis is supported by Lodge’s research in animal models, which shows that loss of GABA-mediated inhibition reduces gamma oscillations and impairs cognitive function [108].

GABA-A receptors are cys-loop ligand-gated chloride/anion channels that generally reduce neuronal electrical excitability and regulate spike timing, thus influencing circuit function [109]. They are responsible for most of the fast synaptic inhibition in the mammalian brain. These postsynaptic receptors are heteropentamers, which, upon binding with GABA released from presynaptic neurons, permit the inward flow of Cl- ions. This influx of anions generates inward currents that temporarily reduce local membrane excitability. A notable characteristic of brain GABA-A receptors is the extensive variety of subunit isoforms that can be incorporated into the receptor heteropentamer. Currently, 16 different subunits, each encoded by distinct genes, have been identified (α1–6, β1–3, γ1, γ2 [short and long splice forms], γ3, δ, ε, π, and θ) [110]. Preclinical studies suggest that blocking the α5 subtype of the GABA receptor (α5-GABAARs) results in behavioral patterns linked to schizophrenia. Additionally, postmortem studies show reduced protein and mRNA levels of α5-GABAARs in the hippocampus of individuals with schizophrenia [100,101]. Marques and his colleagues explored the presence of α5-GABAA receptors in the hippocampus by utilizing PET imaging to compare hippocampal regions between schizophrenic patients and healthy individuals. The findings indicated a decrease in [11C]-Ro15–4513 VT, a radioactive tracer employed in PET scans to measure the total volume of distribution for α5-GABAA receptors, in the hippocampus of untreated schizophrenic patients compared to healthy controls [111].

### 5.5. Glutamate

**Figure 4:**
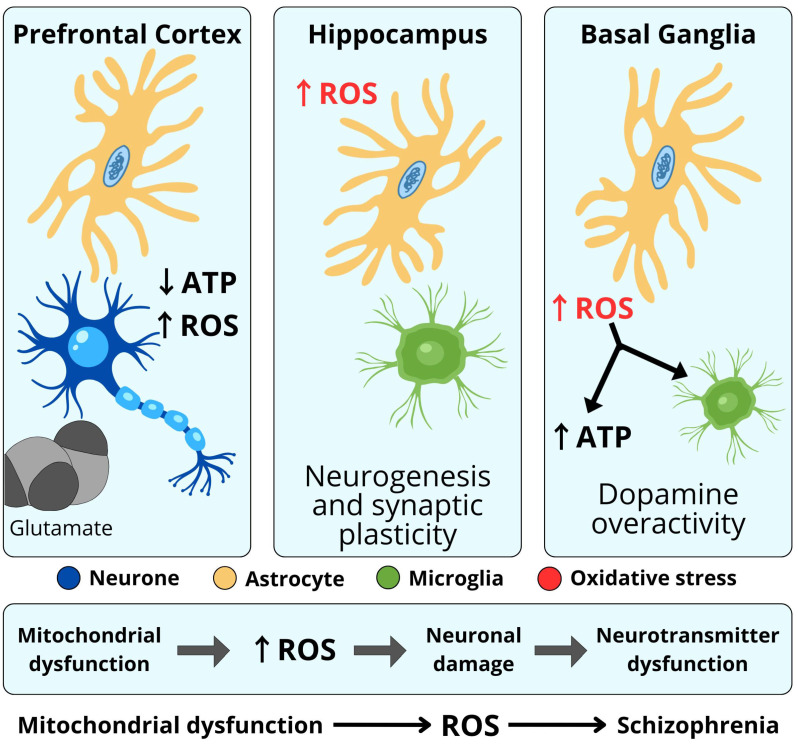
Mitochondrial dysfunction and oxidative stress in the pathophysiology of schizophrenia.

Glutamate is responsible for 50–60% of all neurotransmissions in the brain, while the remaining 40–50% is modulated by GABA-ergic receptors. As a result, 90–99% of neurons are modulated by glutamatergic or GABA-ergic neurotransmission. Thus, functional changes in the synthesis and transmission of these neurotransmitters play a key role in the pathophysiology of schizophrenia (Figure 4) [82,112].

Glutamate is the main excitatory neurotransmitter in the central nervous system, dominating most synapses, but its concentration must be tightly regulated within a narrow physiological range. Pilowsky’s study, using glutamate receptor-binding radionuclides to analyze a model of NMDA-R-hypofunction psychosis, showed reduced NMDA-R binding in the left hippocampus of patients with schizophrenia [113]. In contrast, a study of chronic patients found increased glutamate levels in basal ganglia structures at most stages of the disease (even before antipsychotic treatment) and decreased glutamate levels in the prefrontal cortex [114].

The excitotoxic hypothesis of schizophrenia suggests that progressive neuronal cell death in some cortical areas occurs via over-activation of glutamatergic projections [11]. Assuming that clinical exacerbation of schizophrenia is accompanied by a neuroinflammatory state, leading to cortical neurodegeneration, the levels of glutamate and glutathione (its antioxidant) were verified in selected cortical areas [115]. Using 7T proton magnetic resonance spectroscopy, glutamate and glutathione were significantly decreased in the anterior cingulate cortex (ACC) in accordance with the length of clinical stabilization in schizophrenia. Glutamate excitotoxicity is mediated via nitric oxide, and the inhibition of this degenerative effect is blocked by p38 MAP kinase and c-jun N-terminal kinase i.e., mitogen-activated protein kinases responsive to stress stimuli [116]. Glutamate dysfunction signaling and dysregulation of oxidative stress can be screened even in the early, prodromal phase of schizophrenia, long before its clinically revealed onset [12,117].

### 5.6. Other Neurotransmitters

The contribution of other neurotransmitters to the comprehensive pathogenesis of schizophrenia related to oxygen stress and neuroinflammation concepts is less recognized, but in a general context, the common pathway for some can be focused on such factors as oxygen free radicals, impairing synaptic signaling and brain plasticity mechanisms [118]. The activity of the cholinergic pedunculopontine nuclei (PPN) in schizophrenia is shown to be impaired, leading to the disruption of gamma wave activity and the increased REM sleep drive observed in some patients. The link between the nicotine system and schizophrenia is confirmed by the extremely high prevalence of tobacco dependence, with approximately 65% of patients being smokers [119]. Both post-mortem studies of patients with schizophrenia and PET studies have shown a significant reduction of M1 and M4 receptors in the hippocampus and cerebral cortex, which can be linked to reduced cognitive performance in people with schizophrenia [120,121].

## 6. Neuroinflammation Hypothesis of Schizophrenia

The interaction between the immune system and neuroinflammatory processes plays a critical role in understanding the neurobiology of schizophrenia. Recent studies have indicated that persistent neuroinflammation within the central nervous system may be linked to schizophrenia [120]. For instance, evidence of neuroinflammation has been detected in postmortem brain tissue from individuals with schizophrenia. Despite variations in findings, analyses of gene expression using microarrays on post-mortem brain samples have consistently revealed heightened levels of inflammatory markers like SERPINA3 (a protease inhibitor involved in inflammatory responses and tissue maintenance) and interferon-induced transmembrane protein (IFITM) in patients diagnosed with schizophrenia. In instances of neuroinflammation, hyperactive microglia cells release inflammatory cytokines and reactive oxygen species, leading to the degeneration of neurons, disturbances in white matter integrity, and reduced neurogenesis. This neuronal harm could potentially account for several negative symptoms associated with schizophrenia, including impairments in working memory, cognitive processing speed, and executive functioning [121].

Chemokines serve as immune-regulating proteins that play roles beyond guiding lymphocytes to sites of inflammation. They also contribute to neuromodulation, neurogenesis, and neurotransmission within the nervous system. Studies have demonstrated a genetic link between polymorphisms in chemokine and chemokine receptor genes and schizophrenia [17]. They are categorized into four distinct subfamilies determined by the arrangement and spacing of cysteine residues within their amino acid sequences: CC chemokines (C-C motif chemokine ligands—CCL), CXC chemokines (C-X-C motif chemokine ligands—CXCL), CX3C chemokines (C-X3-C motif chemokine ligands—CX3CL), and C chemokines (C motif ligands—XCL). Robust data, supported by meta-analyses, indicate elevated levels of CXCL8/IL-8, CCL2/MCP-1, CCL4/MIP-1β, and CCL11/eotaxin-1 in the bloodstream of individuals with schizophrenia. While CXCL8 has been identified as increased in cerebrospinal fluid, research on other chemokines in this context is comparatively limited [121].

Among the variants of inflammatory hypotheses, an independent and, above all, one that can be relatively easily tested, is the hypothesis of pro-inflammatory cytokines in the development of the disease, leading to neurodegenerative changes. The effect of inflammation itself is a defensive response of the body’s immune system, equipped with PRR receptors (pattern recognition receptors) to pathogens entering the body. PRRs are associated with a membrane that contains receptors that recognize molecular patterns associated with Toll-like pathogens (TLRs), C-type lectin receptors (CLRs) and other cytosolic receptors. In response to pathogenic proteins entering the body, signaling pathways are activated to neutralize the harmful factor [122]. TRLs diagnose bacterial and viral threats and induce the production of inflammatory mediators, such as cytokines and chemokines. This reaction is initiated by monocytes and lymphocytes (T and B cells), which analyze the incoming antigens (APCs—antigen-presenting cells) and enable the formation of other T cell subgroups responsible for the inflammatory response [123].

As a result of the activation of monocytes, lymphocytes and mast cells, the production of inflammatory cytokines is induced, i.e., IL-1β, IL-6 and TNF-α, responsible for the modulation of the immune reaction and the inflammation itself [124]. Activated phagocytic cells are divided into two groups, M1 and M2 subpopulations. The M1 subpopulation is responsible for the production of pro-inflammatory cytokines (IL-β, IL-6, TNF-α, IL-23 and free oxygen radicals) and the elimination of pathogens (pro-inflammatory role), while M2 is responsible for the production of IL-10 and TGF-β (anti-inflammatory role) [122,123,124]. Neuroinflammation encompasses not only inflammatory processes in the central nervous system (CNS) but also complex interactions between immune, neuronal, and glial cells. Activated microglia and astrocytes modulate neuronal function by releasing cytokines and other signaling molecules that can impair neurogenesis and alter synaptic plasticity. Glial-neuronal interactions contribute to neurodegenerative and psychiatric processes without directly disrupting the blood–brain barrier (BBB), which is influenced by additional oxidative and inflammatory mechanisms, described below [125,126].

Oxidative stress and neuroinflammatory processes disrupt BBB integrity by inducing the production of reactive oxygen species (ROS) and pro-inflammatory cytokines, which impair tight junction proteins and increase BBB permeability [127]. This compromise in BBB function facilitates the infiltration of peripheral immune cells and inflammatory mediators into the CNS, potentially exacerbating neuronal damage and contributing to the pathophysiology of schizophrenia. Clinically, BBB dysfunction may underlie some of the cognitive and behavioral symptoms observed in patients [128].

Microglia also play an important role in the inflammatory process in the CNS. Excessive expression of pro-inflammatory cytokines and increased microglial activity (modification of kynurenine and glutamate signaling) may cause the development of schizophrenia, determined by genetic susceptibility. Moreover, excessive activation of microglia increases the activity of astrocytes, which, through excessive secretion of glutamate, have toxic effects on the CNS and influence neurotransmitter regulation (dopaminergic, serotonergic and glutamatergic). This is confirmed by research related to the impact of inflammation and activation of the immune system in mothers of people with schizophrenia [129,130,131].

An extremely important and important thing in the mutual interaction of microglia and cytokine production is the intensity of the immune response, which undoubtedly influences the process related to the so-called pruning of synapses [131,132]. Pruning synapses enables the creation of new connections through the so-called siding process, and this, in turn, strengthens synaptic connections [133]. The weaker the intensity of the immune response, the more beneficial pruning is. With age, aging microglia are more susceptible to stimuli that trigger the production of pro-inflammatory cytokines and thus reduce synaptic pruning caused by chronic neuroinflammation [134].

Stress also has a negative impact on microglia activation, which promotes inflammation through increased secretion of glucocorticoids and catecholamines. Promoting inflammatory reactions leads to increased release of IL-1β and other pro-inflammatory cytokines, which in turn stimulate the release of glucocorticoids, activating the hypothalamic-pituitary-adrenal axis (HPAA) [135,136,137].

In conclusion, increased production of peripheral inflammatory mediators suggests an association between chronic inflammation and various psychological states and vice versa [137]. The reason for this correlation is probably the disruption of the functioning of various signaling pathways, at various levels, in various diseases, not only immunological, but also psychological [138,139].

## 7. Neurodevelopmental Hypotheses

Genetic predispositions and environmental influences create the two-hit hypothesis, focused on disruptions in normal signaling processes [140,141,142]. Based on adoption, family, and twin studies, at least 15 genetic regions were associated with hereditary component [142]. Being born during the winter or spring months has been linked to an increased risk of schizophrenia, potentially due to maternal nutritional deficiencies or higher rates of viral infections during these seasons [143,144]. Furthermore, a diathesis-stress model of schizophrenia suggests that the condition emerges because of stress exposure impacting a pre-existing vulnerability, which may stem from genetic predispositions or early environmental influences [145,146].

Myelination is a neurodevelopmental process that begins in the third trimester of pregnancy, intensifies during childhood and early adolescence and stabilizes in adulthood [147]. The correct course of myelination requires the proper function of oligodendrocytes at each stage of development. Therefore, oligodendrocyte dysfunction results in disruptions in myelination and neural connectivity, resulting in cognitive deficits at the functional level [148,149]. The control of oligodendrocyte differentiation and myelination processes in the central nervous system is regulated by several pathways such as the phosphatidylinositol-3-phosphate kinase pathway, glycogen synthase kinase 3β or extracellular signal-regulated kinase 1 and 2 (ERK1/2), among others. Damage to oligodendrocytes can occur both at the stage of their differentiation and at the stage of the mature cell. This may be influenced, for example, by an impaired redox balance in oligodendrocyte cells due to high levels of reactive oxygen compounds, lower glutathione concentrations or increased levels of free iron, which directly affects hypomyelination in the prefrontal cortex and hippocampus [82]. In the first phase of schizophrenia, glutathione (GSH) levels correlate with white matter integrity. To confirm the above findings, in vivo proton magnetic resonance spectroscopy was performed, which showed that glutathione levels in the frontal cortex of people with schizophrenia were 52% lower compared to controls [150,151,152]. Furthermore, the study observed that higher glutamate levels in the prefrontal cortex of the amygdala (ACC) were associated with lower intracortical myelin within the prefrontal cortex, particularly when GSH levels in the ACC were low, suggesting an important role for GSH in myelination processes. A study by Spaas et al. [153] conducted on a rat model of cognitive inflexibility associated with schizophrenia showed that the expression of genes related to the antioxidant system was altered throughout the postnatal period and preceded hypomyelination. In addition, reduced glutathione levels and increased mitochondrial numbers were observed. The authors also showed that N-acetylcysteine supplementation had a positive effect on the expression of genes related to myelination processes. Research by Khattar and colleagues suggests a potential link between iron content and myelination. Myelin loss and iron accumulation are key features of neurodegenerative diseases [154]. As a cofactor, iron is an essential component in dendrocytes for numerous enzymes involved in the proliferation and differentiation of oligodendrocyte precursor cells (OPCs), as well as in the synthesis of cholesterol and phospholipids, which are essential components of myelin [155]. Myelin degradation releases large amounts of free iron, which can lead to further demyelination and neurodegeneration. The released iron promotes the formation of amyloid sheets, which exacerbate myelination deficits, resulting in reduced inter-regional connectivity in the central nervous system, which leads to cognitive decline and other CNS function deficits [154]. Iron could also generate toxic free radicals. Oxidative stress, a key factor in myelin damage, is induced by the production of reactive oxygen species and nitric oxide, mainly by microglia and astrocytes. Oxidative damage is exacerbated by the release of iron (II) from degenerating cells into the extracellular space, leading to the formation of highly reactive hydroxyl radicals [155]. In vitro studies clearly indicate that high levels of ROS can induce OPC cell death. Exposure to ROS during OPC differentiation in vitro leads to reduced expression of mature oligodendrocyte and myelin markers, such as GalC and MBP, and altered expression of regulators of cell differentiation. This results in a reduced capacity for myelination in the CNS. Excessive ROS in the early stages of the disease may impair OPC function long before the onset of chronic phases, which are usually associated with impaired remyelination [150].

In summary, myelination depends on the proper functioning of oligodendrocytes, and dysfunction of these cells leads to myelinization disorders and cognitive deficits. We know that factors such as oxidative stress and low glutathione levels, as well as iron accumulation, lead to impairments in this process, providing opportunities for the potential benefits of antioxidant therapies, but at the same time posing several challenges for researchers and clinicians to overcome to successfully introduce these therapies into clinical practice.

The two-hit model is easily “completed” by the action of infectious agents. Animal models have provided substantial evidence indicating the significance of pre- and perinatal infections in the subsequent development of schizophrenia. In these models, offspring exhibit characteristic schizophrenia symptoms, including cognitive decline and abnormalities in startle reflexes, following exposure to viral agents during prenatal stages. Correspondingly, findings from human studies demonstrate associations between prenatal or childhood exposure to various infections such as viral, respiratory, genital, or reproductive tract infections, Toxoplasma gondii, and schizophrenia risk [156]. Notably, animal research has revealed that elevated levels of interleukin 6 (IL-6) during gestation induce schizophrenia-like symptoms in offspring, which can be mitigated by anti-IL-6 antibodies. Furthermore, IL-6 levels during childhood serve as predictive markers for later schizophrenia risk [7,156].

## 8. Antioxidant Therapy

Therapy using antioxidants has the potential to prevent, delay, or ameliorate psychoneurological disorders in general, including schizophrenia [157]. Although these are rather non-specific therapies, such as supplementation of omega-3 unsaturated fatty acids, ascorbic acid and α-tocopherol, they can effectively reduce psychosis symptomatology scores with BPRS or PANSS. However, a meta-analysis of 22 schizophrenia trials showed that the practical possibilities of antioxidant treatment are limited and mostly not relevant to clinicians’ and patients’ needs [158]. Larger trials with longer follow-up periods should be performed. These previous trials have not fully addressed clinical goals (relapse-remission dichotomy), but rather quantitative observations of rating scales. Also, direct use of acetylsalicylic acid, a classical anti-inflammatory drug in the treatment of schizophrenia produced no clear evidence [159]. Trials were too short, too small to draw any rational therapeutic conclusions.

Unlike all approved antipsychotics targeting the D2 dopamine receptor, some of them are believed to be more effective, more neuroprotective, e.g., clozapine, due to their impact on the glutamatergic system, which is believed to reduce neuroinflammation risk [160]. Typical antipsychotics (e.g., haloperidol) stop positive symptoms, but at the same time, during long-term treatment, may elevate extracellular, excitotoxic glutamate, exerting antagonist effects on D2 and 5HT1A receptors. However, direct attempts to develop antipsychotics that specifically target the glutamate neurotransmitter system, mainly by stimulating two receptors called mGluR2 and mGluR3, have not proved to be effective so far, although these drugs (like pomaglumetad) have even reached phase III trials [161].

The sum of unsuccessful studies on agents modulating the glutamate system, e.g., glycine, D-serine and sarcosine, showed that the effectiveness of these drugs may concern approximately 20–30% of patients with schizophrenia and predominant neuroinflammation etiopathogenesis should be considered at least to this extent [116].

The distinction between antipsychotics affecting dopamine and glutamatergic pathways refers to the basic distinction of schizophrenic symptomatology, i.e., positive symptoms, associated with over activity of mostly dopamine pathways, and negative symptoms, associated with cognitive and social deficits, the basis of which is long-term unbalanced oxidative stress, neurotoxicity and neurodegeneration, This, in turn, opens up the possibility of beneficial effects of stimulation of neuroprotective and neurodevelopmental factors [162].

## 9. Oxidative Stress and Neuroinflammation in General Models of Schizophrenia

All modern models of schizophrenia can be essentially supplemented or mostly explained by the core oxidative stress and neuroinflammation [163]. Due to this complementarity, oxidative stress and neuroinflammation concepts can be easily included to the unitary super-hypothesis of schizophrenia [120].

- Neurodevelopmental models. Regardless of whether these models approach schizophrenia as an inevitable cost to the emergence of the human species or as a variable sum of errors at key stages of an individual’s development, they address the interplay of unimpaired development and pathological processes such as oxidative stress and neuroinflammation [164,165].
- The vulnerability-stress models. The phenomena of oxidative stress and neuroinflammation may be particularly useful for explaining not only the initial or final psychotic pathogeneses, but mostly the “silent”, long-term accumulating dysfunctions of the prodromal and latent psychotic phases [120,141].
- Neuroplasticity models. Oxidative stress and neuroinflammation concepts allow us to avoid understanding schizophrenia through a single trigger or disruption of a key stage of development and to see the pathogenesis of psychosis as an imbalance of continuous pro-health and disruptive neuroplastic processes, such as apoptosis induced by oxidative stress.
- Neurotransmitter models. All these concepts, such as early hyperdopaminergic or late hypodopaminergic hypotheses, refer to quantitative making qualitative changes in neurotransmitter functioning and are finally related to cumulative damage to the CNS [29,60,100,108]. While the list of primary causes damaging the CNS is quite long or in fact endless, the secondary reasons such as oxidative stress and neuroinflammation are limited.
- Environmental models. All environmental models, both biological (e.g., viral hypothesis) and biochemical (e.g., toxic hypothesis), focus on oxidative stress and consequently neuroinflammation as a common part of these concepts [129,140,162].
- Microbiome-brain axis models. Oxidative stress and inflammation may contribute to the disruption of the intestinal and blood–brain barriers, and ultimately brain inflammation [60].
- Psychological stress models. All concepts of psychological stress use oxidative and neuroinflammatory dysfunctions as a common link between non-compensated stress and neuropathological consequences [137,138,139]. When stressful life events exceed an individual’s neuroplasticity buffering threshold, a psychotic episode is likely to develop [134].
- Factor models. Because there is a relative independence of factors creating clinical symptoms of schizophrenia (positive, negative, cognitive, disorganized, emotional, others), non-specific oxidative stress and neuroinflammation can explain the individual diversity of clinical symptoms of schizophrenia and varying susceptibility to antipsychotic treatment [5,140,162].
- Stress and neuroinflammation models. Stress and neuroinflammation, constituting—both combined or independently—such a comprehensive and fundamental sum of concepts, can create in fact their own models of schizophrenia [119,121,134].
- To provide a broader perspective on the mechanisms discussed, Table 2 compiles and synthesizes key clinical and preclinical findings that demonstrate a consistent association between redox dysfunction and schizophrenia.

## 10. Conclusions

The concepts linking oxidative stress and schizophrenia are based both on specific and non-specific mechanisms. The oxidation reaction is a fundamentally natural process that can converge into oxidative stress under the influence of genetic and environmental factors. However, the oxidative stress, as a candidate for the convergence point or central hub of the etiopathogenesis of schizophrenia, still requires conceptualization. Studies indicate that elevated levels of oxidative markers and pro-inflammatory cytokines are commonly observed in people with schizophrenia, indicating a potential pathophysiological link. Dysregulation of redox homeostasis can exacerbate neuroinflammatory responses, contributing to neuronal damage and subsequent manifestation of psychiatric symptoms. The present study showed that oxidative stress causes several changes, such as:

(1) mitochondrial dysfunction, abnormal neuron formation, abnormal myelination, impaired neurotransmitter production, and impaired reorganization of synaptic connections,
(2) impaired release of neurotransmitters, abnormal processes of neurogenesis,
(3) a deficit of ATP and a disturbed energy balance of cells and their enzymatic activity,
(4) lipid peroxidation, altered protein structure, mutations in protein coding, disturbed protein–protein interaction,
(5) DNA mutations, impaired gene expression, and impaired activation of DNA repair pathways,
(6) disrupted cell homeostasis as a toxic effect of high H_2_S concentration,
(7) destruction of metabolic pathways and changes in substrate utilization,
(8) increased apoptosis and increased inflammatory responses,
(9) microdamage of dopaminergic endings and loss of neuronal connections,
(10) impaired synthesis and transmission of neurotransmitters,
(11) impaired synaptic signaling,
(12) non-enzymatic oxidation of dopamine—an increase in hydrogen peroxide,
(13) increased levels of nitric oxide (peroxynitrite)—neurotoxic effects.

In this regard, various models of biochemical cascades, long-term cumulative processes or feed-forward loops of increasing adverse effects are proposed.

However, there are no properly designed studies in this area that combine both clinical and biochemical assessment. The access to new neuroimaging techniques with biochemical assessment may soon change this situation [114,120].

Further research should identify specific biomarkers in homogenous groups of patients, operationalizing the impact of oxidative stress, the modification of which can enable effective therapy in schizophrenia.

## Figures and Tables

**Table 2:** Summary of Key Experimental and Clinical Evidence Linking Redox Imbalance and Oxidative Stress to Schizophrenia Pathophysiology.

Type of Evidence	Brief Summary/Significance	Ref. No.
Humans—clinical (biochemistry)	Demonstrated disturbed redox balance (reduced GSH) in patients with schizophrenia—one of the most frequently cited studies linking antioxidant deficit with schizophrenia.	Yao et al., 2006 [47]
Humans—7T MRS (imaging)	7-Tesla MRS revealed alterations in glutathione/glutamate levels in patients’ brains, providing direct evidence of redox dysfunction in brain tissue.	Kumar et al., 2020 [115]
In vitro—neurons	Experiments showing that dopamine, under glutathione deficiency, induces oxidative stress in neurons—a mechanistic explanation for the DA-ROS relationship.	Grima et al., 2003 [84]
Mixed—post-mortem humans + MIA mice	Increased expression of immune transcripts in the midbrain of patients and in the MIA model, linking neuroinflammation with developmental disorder models.	Purves-Tyson et al., 2019 [134]
Humans—post-mortem transcriptomics	Broad upregulation of inflammatory markers in the PFC/striatum/hippocampus in post-mortem samples from schizophrenia patients.	Lanz et al., 2019 [142]
Animals—neurodevelopmental rat model	Aripiprazole + NAC intervention normalizes cysteine-related disturbances and “schizophrenia-like” behaviors—preclinical evidence that redox modulation has behavioral effects.	Górny et al., 2023 [45]
In vitro/ex vivo—human fibroblasts	Pilot study on fibroblasts showing apoptotic shifts in schizophrenia patients—cellular evidence of mitochondrial dysfunction/apoptosis.	Catts et al., 2006 [62]
In vitro/biochemistry	Identification of mitochondrial ROS generation sites for different substrates—mechanistic basis linking mitochondrial dysfunction with increased ROS production.	Quinlan et al., 2013 [38]
In vitro/OPC	Oxidative stress disrupts differentiation of oligodendrocyte precursor cells—a link between ROS and abnormal myelination, relevant to schizophrenia pathology.	Spaas et al., 2021 [153]

## Data Availability

No new data were created or analyzed in this study.
